# Supplementary material for: Evaluation of total immunoglobulin G and subclass antibodies in an enzyme-linked immunosorbent assay for serodiagnosis of human amebic liver abscess
Source: PeerJ. 2022 Sep 29;10:e14085. doi: 10.7717/peerj.14085 (PMC9527022; doi:10.7717/peerj.14085)
Supplement: Supplemental Information 2 [file peerj-10-14085-s002.docx]

**Table S1:** **Demographic and clinical information of Japanese amebic liver abscess (ALA) patients examined in this study.**

| No. | Patient’s demographic data (sex, age) | Type | Clinical courses, diagnosis and treatment |
| --- | --- | --- | --- |
| 1 | Male, N/A | Liver abscess | No detail information on imaging examinations, but serologically positive against *E. histolytica* antigen (ELISA > 800, IHA > 160, IFA > 800, Ouchterlony +). |
| 2 | Male, 24 | Liver abscess | Fever and right hypochondrial pain presented in October 1983; liver abscess (13 cm in diameter) diagnosed by CT; Serologically positive against *E. histolytica* antigen (ELISA > 6400, Ouchterlony +). Treated with dehydroemetine. Stayed in Bangladesh for 20 months before onset. |
| 3 | Male, 44 | Liver abscess and colitis | Diagnosed as ALA by biopsy of liver abscess in September 1993; Serologically positive against *E. histolytica* antigen (ELISA > 1600, Ouchterlony +). C-reactive protein (CRP) 17; *E. histolytica* cysts also detected in the stool. Blood sampling was done at the time of consultation. Homosexual suspected. |
| 4 | Female, 50 | Liver abscess | Fever (39℃) and right hypochondrial pain presented in April 1991. Abscess (10 x 4 cm) in left lobe (S3) detected by CT and US; Serologically positive against *E. histolytica* antigen (ELISA > 6400, IHA +, Ouchterlony +). CRP 6+; Bacterial abscess negative. |
| 5 | Male, 59 | Liver abscess | No detail information on imaging examination, but serologically positive against *E. histolytica* antigen (ELISA > 800, IHA > 80, IFA > 200, Ouchterlony +). |
| 6 | Male, N/A | Liver abscess | No detail information on imaging examination is recorded, but liver abscess diminished and inflammation improved in January 1994. The timing of blood sampling done at the time of consultation. Serologically positive against *E. histolytica* antigen (Ouchterlony +). |
| 7 | Male, N/A | Liver abscess | Liver abscess diagnosed in 1980, but no detail information is available. Serologically positive against *E. histolytica* antigen (ELISA > 12800, IHA > 20480, IFA > 3200, Ouchterlony +). |
| 8 | Male, 27 | Liver abscess | Right hypochondrial pain presented on February 8, 1997; fever (38–39℃) on February 17, 1997; solitary abscess (3–4 cm in diameter) in the hepatic left lobe by CT and US on February 20, 1997. Blood sampling was done on February 22 at the time of consultation at Juntendo University Hospital. Serologically positive against *E. histolytica* antigen (ELISA > 400, Ouchterlony + on February 25, 1997. First diarrhea, not mucous/bloody stool, presented during staying in Mexico in March­–April, 1996. |
| 9 | Male, 60 | Liver abscess | Fever (39.5℃) and fatigability presented in June 1992. Bacterial abscess suspected, but not effective to antibiotics. As anamnesis, multiple ulcers in cecum and rectum revealed by colonoscopy in 1991, so ALA suspected. SOL in right lobe S5, S6 and S7 detected by US and CT. Blood sampling at 4 days after fever, and serologically positive against *E. histolytica* antigen (ELISA > 1600, Ouchterlony +). Cured with metronidazole. |
| 10 | Male, 33 | Liver abscess | Fever and right hypochondrial pain presented in April 1984. Liver abscess (8 cm in diameter) by US and CT; Blood test, CRP 6＋; Serologically positive against *E. histolytica* antigen (ELISA >25600, Ouchterlony +, CF test +, IHA +. Homosexual history (6–7 years). |
| 11 | Male, 46 | Liver abscess | Fever and abdominal pain presented in November 1992. Multiple liver abscesses in S3, S5, S8 detected by CT and US. Serologically positive against *E. histolytica* antigen (ELISA>400, Ouchterlony +). Homosexual suspected. |
| 12 | Male, 60 | Liver abscess | Diarrhea presented in August 1982, and watery diarrhea lasted until September. Diarrhea reoccurrence in February 1983. Fever (37–38℃) in January and 38–40℃ in February. In March 1983 right hypochondrial pain presented, cholecystitis suspected and hospitalized. At admission, a giant liver abscess detected by US and drained (purulent >500 ml). First partial hepatectomy done on June 15, 1983 and postoperative process went smoothly. However, January 1988 (5 years later), fever (38.2℃) recurrence. CRP 6+. A giant liver abscess in S7 (12.5 cm in diameter) by CT; Bacterial abscess negative. Fever and liver abscess reoccurrence, and hospitalized on June 18, 1988. Second partial hepatectomy done on June 24, 1988 and drained, but fever and right hypochondrial pain reoccurrence on July 27 and liver abscess re-diagnosed by CT. Nevertheless, repeated fever reoccurrence and abnormal lesions extended in both lungs by chest X-ray. No effect with antibiotics. On October 27, 1993, ALA diagnosed at Juntendo University Hospital, and finally cured with metronidazole. Serologically positive against *E. histolytica* antigen (ELISA > 12800, Ouchterlony +). He possibly got infected during a couple of day tours in Bangkok, Thailand, in March 1981. |
| 13 | Male, 30 | Liver abscess | Fever (38℃) and diarrhea presented in February, 1992; Fever improved by antibiotics, but bloody stool lasted until on February 15. Chronic proctitis suspected by colonoscopy on March 5, but bloody stool not improved by sarazopyrin. Fever (37.8℃) reoccurrence on May 4, but not effective against antibiotics. Liver abscess (4 cm in diameter) in S6 diagnosed by US at Juntendo University Hospital on May 25. Drained on March 6. Blood sampling at 3 months after onset (May 25 at the time of consultation). Serologically ALA suspected (ELISA > 800, Ouchterlony +) on May 29. Cured with metronidazole. |
| 14 | Male, 56 | Liver abscess and colitis | Diarrhea and right hypochondrial pain presented on 5 and 10 October 1992; multiple liver abscesses by CT on November 18. Diarrhea became mucous/bloody stool on November 15 and multiple ulcers in sigmoid colon diagnosed by sigmoid colon endoscope. Elevated ALP (503 IU/L) and GGTP (127 IU/L); Serologically positive against *E. histolytica* antigen (Ouchterlony +, ELISA +, titer not available). Cured with metronidazole. |

N/A = not available.
